# Lactic acid bacteria and yeast strains isolated from fermented fish (Budu) identified as candidate ruminant probiotics based on *in vitro* rumen fermentation characteristics

**DOI:** 10.14202/vetworld.2023.395-402

**Published:** 2023-02-28

**Authors:** Laily Rinda Ardani, Yetti Marlida, Mardiati Zain, Jamsari Jamsari, Dilla Mareistia Fassah

**Affiliations:** 1 PMDSU Program, Graduate Program, Faculty of Animal Science, Andalas University, Padang, West Sumatera, Indonesia; 2 Department of Animal Nutrition, Faculty of Animal Science Andalas University, Padang, West Sumatera, Indonesia; 3 Department of Plant Breeding, Faculty of Agriculture, Andalas University, Padang, West Sumatera, Indonesia; 4 Department of Nutrition and Feed Technology, Faculty of Animal Science, IPB University, Bogor, West Java, Indonesia

**Keywords:** *in vitro*, lactic acid bacteria, probiotics, rumen fermentation, yeast

## Abstract

**Background and Aim:**

Probiotic supplementation can assist with manipulating the rumen microbial ecosystem. Lactic acid bacteria and yeast from fermented fish (Budu) as the indigenous food from West Sumatra, Indonesia, are potential probiotics for livestock. This study aims to select the best candidate lactic acid bacteria and yeast strains from fermented fish as ruminant probiotics and evaluate the effect of their supplementation on the characteristics of rumen fermentation, feed digestion, and total gas production *in vitro*.

**Materials and Methods:**

This study used nine treatments, performed in triplicate, in a completely randomized design. The substrate ratio comprised of 70% *Pennisetum purpureum* forage and 30% concentrate. Five lactic acid bacteria and three yeast isolates were used in this study. Treatments were as follows: T0: control (basal diet); T1: T0 + *Lactobacillus parabuchneri* strain 3347; T2: T0 + *Lactobacillus buchneri* strain 5296; T3: T0 + *Lactobacillus harbinensis* JCM 16178; T4: T0 + *Schleiferilactobacillus harbinensis* strain LH991; T5: T0 + *L. parabuchneri* strain 6902; T6: T0 + *Pichia kudriavzevii* strain B-5P; T7: T0 + *P. kudriavzevii* strain CBS 5147; and T8: T0 + commercial yeast (*Saccharomyces cerevisiae*). The lactic acid bacteria inoculum contained 1.02 × 10^11^ colony-forming unit (CFU)/mL, while the yeast inoculum contained 1.5 × 10^10^ CFU/mL.

**Results:**

The results showed that four lactic acid bacteria and three yeast produced a higher total gas yield (104–183.33 mL) compared to the control (103 mL). Supplementation with lactic acid bacteria in the rumen fermentation *in vitro* showed dry matter digestibility of 63%–70% and organic matter digestibility (OMD) of 64%–71%. We observed that total volatile fatty acid (VFA) production in all treatments was significantly higher (86–121 mM) compared to the control (81 mM). The concentration of NH_3_ production was higher in all treatments (12.33–16.83 mM) than in the control (12.25 mM). Meanwhile, the probiotic supplementation did not cause a significant change in the rumen pH (6.86–7.12). Supplementation with the lactic acid bacteria *S. harbinensis* strain LH991 consistently demonstrated the best results from the parameters of dry and OMD (70.29% and 71.16%, respectively), total VFA (121.67 mM), NH_3_ (16.83 mM), and total gas production (149.17 mL). The best results were observed from the yeast candidate *P. kudriavzevii* strain B-5P, where the results were dry and OMD (67.64% and 69.55% respectively), total VFA (96.67 mM), NH_3_ (13.42 mM), and total gas production (183.33 mL).

**Conclusion:**

Based on the obtained results, lactic acid bacteria *S. harbinensis* strain LH991 and yeast *P. kudriavzevii* strain B-5P are attractive candidates to be utilized as probiotics for ruminants based on their potential to improve rumen fermentation *in vitro*. This probiotic supplementation can increase the digestibility of feed ingredients, production of total VFA and NH_3_, and total gas produced.

## Introduction

Increasing the productivity of ruminant livestock can be achieved by manipulating the rumen microbial ecosystem. Supplementation with live microbes as probiotics is a safe and feasible alternative to replace antibiotics because they do not cause toxicity in livestock products and leave no residue [1]. Probiotics are beneficial live microorganisms that, when administered in sufficient quantities, provide health benefits to the host [2]. The use of probiotics in animal husbandry contributes to the balance of microbiota activity in the gastrointestinal tract [3], productivity and health of dairy cows [4], host immune function [5], and increased milk production and yield [6]. In addition, the general health benefits of probiotic supplementation of the digestive system in ruminants include the reduction of methanogenesis, control of acidosis, improves digestion, encourages growth of the rumen and intestinal epithelium, and increases nutrient absorption [7].

Seo *et al*. [8] reported that microorganisms commonly used as probiotics for ruminants originate from various genera, such as *Lactobacillus*, *Streptococcus*, *Bacillus*, *Bifidobacterium*, *Enterococcus*, and *Propionibacterium*. The yeast products commonly used include *Saccharomyces* and *Aspergillus* [8]. Nuraida [9] explored various lactic acid bacteria from Indonesian fermented foods, which have characteristics to be potential probiotics. Anggraini *et al*. [10] isolated the lactic acid bacteria from fermented foods native to West Sumatra, including fermented fish, buffalo milk (*dadih)*, durian, and cassava. Harnentis *et al*. [11] also researched the probiotic potential of lactic acid bacteria derived from the fermented foods native to West Sumatra, such as curd, tape, and Budu fish. Both studies demonstrated that lactic acid bacteria isolated from fermented foods native to West Sumatra have the potential to be used as livestock probiotics. Other fermented food products, such as tempeh, have also been reported to contain yeast and lactic acid bacteria [12]. One of the fermented fish products, namely Budu, is made from larger marine fish and is mainly produced in the Pasaman area, about 300 km from Padang, the capital city of West Sumatra [13]. Leatherskin (*Chorinemus* spp.) and Spanish mackerel (*Scomberomorus* spp.), locally known as Ikan Talang and Ikan Mackerel, respectively, are the main fish used to make Budu [13]. The bacteria involved in fish fermentation may include *Lactobacillus* spp., *Micrococcus* spp., *Flavobacterium* spp., *Staphylococcus* spp., *Streptococcus* spp., *Pediococcus* spp., and *Pseudomonas* spp. [13] The lactic acid bacteria and yeast from fermented fish (Budu) have the potential as probiotics for ruminants. Probiotics, especially the beneficial bacteria and yeast, can restore the microbial balance of the digestive tract and against pathogenic bacteria [14]. Lactic acid bacteria can interact with the rumen microorganisms, increase propionate and total volatile fatty acid (VFA) production, enhance feed efficiency and growth performance, and reduce methane gas production [15] and the incidence of diarrhea [16]. Meanwhile, yeast supplementation in livestock rations can increase productivity, health, use of cellulose material, and reproduction [17, 18]. Yeast can reduce oxygen accumulation, prevent the overproduction of lactic acid, and normalize fermentation in the rumen [8]. One type of yeast, *Saccharomyces cerevisiae* can produce metabolites as growth factors, such as vitamins or organic acids, which stimulates the population of lactic acid utilizing bacteria rumen lactate and cellulolytic bacteria [8]. The combination of *S. cerevisiae* and rumen microbes can increase the population of rumen bacteria and fermentability and reduce the acetate: propionate ratio [19].

The increased productivity and performance of livestock through the addition of lactic acid bacteria and yeast in ruminants show varied data. Different strains affect the ability of the probiotics to increase rumen fermentation [1]. The effect of various strains in the addition of lactic acid bacteria and yeast to rumen fermentation requires further investigation. Therefore, it is important to select the best candidate strains of lactic acid bacteria and yeast, which have beneficial effects on rumen fermentation. This study aims to identify the best candidate of lactic acid bacteria and yeast strains from fermented fish (Budu) originating from West Sumatra, Indonesia as ruminant probiotics and evaluate their supplementation effect on the rumen fermentation, feed digestion, and total gas production characteristics *in vitro*.

## Materials and Methods

### Ethical approval

Ethical approval was not required as this was an *in vitro* study. Goat rumen fluid was collected from the slaughterhouse.

### Study period and location

This *in vitro* study was conducted from August to October 2022 at the Feed Industry Technology Laboratory, Non-Ruminant Nutrition Laboratory, Ruminant Nutrition Laboratory, and Animal Biotechnology Laboratory, Faculty of Animal Sciences, Andalas University, Padang, West Sumatra, Indonesia. Observation of total gas production and analysis of rumen characteristics and digestibility of feed ingredients were carried out at the Ruminant Nutrition Laboratory and Non-Ruminant Nutrition Laboratory, Faculty of Animal Science, Andalas University.

### Culture conditions

Five lactic acid bacteria isolates (*Lactobacillus parabuchneri* strain 3347, *Lactobacillus buchneri* strain 5296, *Lactobacillus harbinensis* JCM 16178, *Schleiferilactobacillus harbinensis* strain LH991, and *L. parabuchneri* strain 6902) and two yeast isolates (*Pichia kudriavzevii* strain B-5P and *P. kudriavzevii* strain CBS 5147) from fermented fish (Budu) were obtained the collection of the Feed Industry Technology Laboratory, Faculty of Animal Science, Andalas University. Another isolate, the yeast *S. cerevisiae*, was obtained from commercial yeast (Fermipan, PT Sangra Ratu Boga, West Jakarta, Indonesia). Inoculums of lactic acid bacteria were immunized in 10 mL of DeMan Rogosa Sharpe Broth medium (Merck, Darmstadt, Germany) and incubated at 37°C for 24–48 h under anaerobic conditions. Yeast inoculums were grown in 10 mL of liquid Yeast Peptone Dextrose media with the following ingredients: 2 g glucose (Merck KGaA, Darmstadt, Germany, CAS-No: 50-99-7), 1 g yeast extract powder (HiMedia Laboratories Pvt. Ltd., India), and 2 g buffered peptone water (Merck KGaA). The liquid media inoculated with the yeast isolates were incubated for 24–48 h at 35°C–37°C.

### Experimental design

The goat rumen fluid that was used in this study was obtained from a slaughterhouse in Padang, West Sumatra, Indonesia which was regulated by a completely randomized design 9 × 3 of nine treatments performed in triplicate. The substrate consisted of 70% *Pennisetum purpureum* forage and 30% concentrate. The composition of the rations and nutritional content used in this study is shown in Table-1 [20]. The eight isolates used consisted of five lactic acid bacteria and three yeast isolates. The treatments were as follows: T0: control (basal diet); T1: T0 + *L. parabuchneri* strain 3347; T2: T0 + *L. buchneri* strain 5296; T3: T0 + *L. harbinensis* JCM 16178; T4: T0 + *S. harbinensis* strain LH991; T5: T0 + *L. parabuchneri* strain 6902; T6: T0 + *P. kudriavzevii* strain B-5P; T7: T0 + *P. kudriavzevii* strain CBS 5147; and T8: T0 + commercial yeast (*S. cerevisiae*). The lactic acid bacteria inoculum contained 1.02 × 10^11^ colony-forming unit (CFU)/mL and the yeast inoculum contained 1.5 × 10^10^ CFU/mL. The inoculum dose of lactic acid bacteria and yeast used was 6.6 mL. The McDougall’s buffer solution was made with NaHCO_3_ 9.8 g (Merck KGaA, CAS-No: 144-55-8), Na_2_HPO_4_.7H_2_O 3.68 g (Merck KGaA, CAS-No: 10028-24-7), KCl 0.57 g (Merck KGaA, CAS-No: 7447-40-7), MgSO_4_.7H_2_O 0.12 g (Merck KGaA, CAS-No: 10034-99-8), NaCl 0.47 g (Merck KGaA, CAS-No: 7647-14-5), CaCl_2_ 0.47 g (Merck KGaA), and filled up to 1000 mL with distilled water. The rumen contents were squeezed out and filtered through a double layer of sterile gauze and transferred to the sterile tube in a water bath.

**Table-1:** Ingredients and chemical composition of the rations.

Item	Content
Ingredients (%)	
*Pennisetum purpureum*	70
Cassava waste	15
Tofu waste	6
Soybean	8
Mineral premix^a^	1
Chemical composition (%)	
Dry matter	90.45
Organic matter	90.12
Crude protein	13.00
Crude fiber	22.81
Crude fat	3.13
Ash	9.88
BETN	41.63
TDN^b^	63.58

a Mineral premix (Ministry of Agriculture, the Republic of Indonesia No. D. 2007655678): Composition per kilogram contains calcium carbonate 500 g, phosphate flour 150 g, manganese sulfate 1.25 g, potassium iodide 250 g, cuprum sulfate 0.7 g, sodium chloride 50 g, ferrous sulfate 2 g, zinc oxide 1 g, magnesium sulfate 60 g.

b TDN was calculated based on the Sutardi formula [20]. BETN=Nitrogen-free extract, TDN=Total digestible nutrient

The substrate ration (2.5 g) (Table-1) was put into a 300 mL capacity Erlenmeyer bottle and filled with 50 mL of rumen fluid, 200 mL of McDougall’s solution, and 6.6 mL (2.64%) of the inoculum. The Erlenmeyer bottle was closed with a rubber cap and an anaerobic condition was induced with the flow of CO_2_ gas for about ±2 min. The bottle was incubated in a shaker incubator at 39°C and 1006× *g* for 48 h. After *in vitro* fermentation, the samples were centrifuged at 1509× *g* for 30 min at 4°C. The supernatant/liquid fraction results were used for VFA and NH_3_ analysis. The supernatant was stored at −20°C until it was used to analyze the VFA and NH_3_. The residue was filtered using filter paper (Whatman™ 41; CAT No 1441-125, China) and dried at 60°C for 24 h. The residue or solid part was used to measure the dry and organic matter’s digestibility. *In vitro* dry matter digestibility (DMD) and *in vitro* organic matter digestibility were calculated based on the previously reported Tilley and Terry method [21], using the formula:


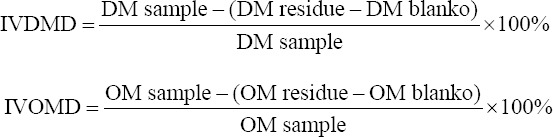


### Parameter measurements

Total gas production was measured during the 48 h incubation using a 60 mL syringe. The syringe needle was injected into the gas holder attached to the erlenmeyer bottle. Readings during incubation were carried out quickly to avoid changes in temperature. Total gas production was measured during the 48 h incubation using a 60 mL syringe. Incubation was stopped by placing the Erlenmeyer bottle into an ice water bath to halt microbial activity. Rumen pH was calculated using a pre-calibrated pH meter. The concentration of NH_3_ was calculated using the previously reported Conway micro diffusion method [22]. Measurement of the total VFA concentration was performed using the previously reported steam distillation method [23]. The residue was used to analyze the dry and organic matter’s digestibility using the previously reported proximate analysis method [21].

### Statistical analysis

Observational data were analyzed using a one-way analysis of variance. Data showing significantly different results (p < 0.05) was confirmed by the least significant difference test. Differences between the treatments we further analyzed using IBM Statistical Package for the Social Sciences Statistics 26.0 version (IBM Corp., NY., USA).

## Results

Probiotic supplementation of five lactic acid bacteria isolates (*L. parabuchneri* strain 3347, *L. buchneri* strain 5296, *L. harbinensis* JCM 16178, *S. harbinensis* strain LH991, and *L. parabuchneri* strain 6902), two yeast isolates *P. kudriavzevii* strain B-5P and *P. kudriavzevii* strain CBS 5147 was selected from fermented fish (Budu) and one commercial yeast isolate containing *S. cerevisiae* as ruminant probiotic candidates. These underwent *in vitro* fermentation and the rumen fermentation products (Table-2) and different digestibility were evaluated (Table-3).

**Table-2:** Rumen fermentation characteristics.

Treatments	pH	VFA total (mM)	NH_3_ (mM)
T0	6.92 ± 0.12	81.67^a^ ± 2.89	12.25^a^ ± 0.25
T1	6.86 ± 0.02	91.67^de^ ± 2.89	13.50^bc^ ± 0.00
T2	7.05 ± 0.17	101.67^h^ ± 2.89	12.50^a^ ± 0.25
T3	7.03 ± 0.26	90.00^d^ ± 5.00	12.33^a^ ± 0.38
T4	6.86 ± 0.02	121.67^i^ ± 2.89	16.83^e^ ± 0.289
T5	6.88 ± 0.03	98.33^fg^ ± 2.89	15.25^d^ ± 0.25
T6	7.09 ± 0.01	96.67^f^ ± 2.89	13.42^b^ ± 0.29
T7	6.98 ± 0.04	86.67^b^ ± 2.89	12.42^a^ ± 0.14
T8	6.90 ± 0.09	88.33^bc^ ± 2.89	12.58^a^ ± 0.29
SEM	0.024	2.225	0.296

VFA=Volatile fatty acid, T0: Control (basal diet); T1: T0 + *Lactobacillus parabuchneri* strain 3347; T2: T0 + *Lactobacillus buchneri* strain 5296; T3: T0 + *Lactobacillus harbinensis* JCM 16178; T4: T0 + *Schleiferilactobacillus harbinensis* strain LH991; T5: T0 + *Lactobacillus parabuchneri* strain 6902; T6: T0 + *Pichia kudriavzevii* strain B-5P; T7: T0 + *Pichia kudriavzevii* strain CBS 5147; T8: T0 + commercial yeast (*Saccharomyces cerevisiae*). Superscripts ^a,b,c,d,e^mean significantly different in a column (p < 0.05), SEM=Standard error of the mean

### Rumen fermentation

Supplementation with the various strains of lactic acid bacteria and yeast sourced from fermented fish (Budu) and commercial yeast did not significantly change the rumen pH (p > 0.05) (Table-2). Total VFA production was significantly increased (p < 0.05) by the supplementation with lactic acid bacteria and yeast (Table-2). The control (basal diet) produced the lowest total VFA (81.67 mM) than the other treatments. The highest VFA produced by the lactic acid bacteria candidates was produced by the T4: supplementation with *S. harbinensis* strain LH991 (121.67 mM) and yeast candidates by the T6: supplementation with *P. kudriavzevii* strain B-5P (96.67 mM). NH_3_ concentration was significantly increased (p < 0.05) by the addition of the lactic acid bacteria and yeast compared to the control (Table-2). Treatment without probiotics (control) resulted in the lowest NH_3_ production of 12.25 mM. These results were not significantly different from supplementation with T2: *L. buchneri* strain 5296 (12.50 mM), T3: *L. harbinensis* JCM 16178 (12.33 mM), T7: *P. kudriavzevii* strain CBS 5147 (12.42 mM), and T8: commercial yeast isolate *S. cerevisiae* (12.58 mM). The highest NH_3_ production was from supplementation with T4: *S. harbinensis* strain LH991 (16.83 mM) for lactic acid bacteria candidates and T6: *P. kudriavzevii* strain B-5P (13.42 mM) for yeast candidates.

### Digestibility

The digestibility of dry and organic matter was significantly increased (p < 0.05) by the supplementation with lactic acid bacteria and yeast from fermented fish (Budu) (Table-3). Two of the five lactic acid bacteria isolates used in the *in vitro* rumen fermentation that is the supplementation with T4: *S. harbinensis* strain LH991 and T5: *L. parabuchneri* strain 6902 resulted in significantly higher dry and organic matter digestibility (OMD) than the other treatments. The control showed 63.40% and 64.36% digestibility of dry and organic matter, respectively. Supplementation with T4: *S. harbinensis* strain LH991 showed the highest dry and OMD with 70.29% and 71.16%, respectively, which were significantly different from other treatments. The addition of the three yeast isolates indicated a higher digestibility value of dry and organic matter than the control. Supplementation with T6: *P. kudriavzevii* strain B-5P showed the highest dry and OMD between the yeast candidates, with 67.64% and 69.55%, respectively. These results were significantly different compared to the digestibility when supplemented with T7: of *P. kudriavzevii* strain CBS 5147 (64.08% and 65.73%, respectively), and T8: commercial yeast *S. cerevisiae* (64.32% and 64.82%, respectively).

**Table-3:** *In vitro* digestibility.

Treatments	DMD (%)	OMD (%)
T0	63.40^a^ ± 1.78	64.36^a^ ± 2.08
T1	63.05^a^ ± 1.44	64.06^a^ ± 1.39
T2	63.89^a^ ± 2.04	64.98^a^ ± 2.28
T3	64.72^a^ ± 3.53	65.40^a^ ± 3.28
T4	70.29^d^ ± 0.53	71.16^cd^ ± 0.48
T5	65.65^b^ ± 0.91	67.28^ab^ ± 0.77
T6	67.64^bc^ ± 0.49	69.55^c^ ± 1.56
T7	64.08^a^ ± 3.03	65.73^a^ ± 3.34
T8	64.32^a^ ± 1.64	64.82^a^ ± 1.73
SEM	0.543	0.567

DMD=Dry matter digestibility, OMD=Organic matter digestibility, T0: Control (basal diet); T1: T0 + *Lactobacillus parabuchneri* strain 3347; T2: T0 + *Lactobacillus buchneri* strain 5296; T3: T0 + *Lactobacillus harbinensis* JCM 16178; T4: T0 + *Schleiferilactobacillus harbinensis* strain LH991; T5:T0 + *Lactobacillus parabuchneri* strain 6902; T6: T0 + *Pichia kudriavzevii* strain B-5P; T7: T0 + Pichia kudriavzevii strain CBS 5147; T8: T0 + commercial yeast (*Saccharomyces cerevisiae*). Superscripts ^a, b, c, d, e^ mean significantly different in a column (p<0.05). SEM=Standard error of the mean

### Total gas production

**Figure-1:**
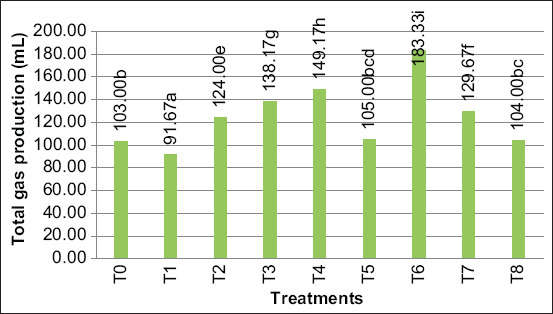
Total gas production by *in vitro* rumen fermentation during 48 h of incubation, T0: control (basal diet); T1: T0 + *Lactobacillus parabuchneri* strain 3347; T2: T0 + *Lactobacillus buchneri* strain 5296; T3: T0 + *Lactobacillus harbinensis* JCM 16178; T4: T0 + Schleiferilactobacillus harbinensis strain LH991; T5: T0 + *Lactobacillus parabuchneri* strain 6902; T6: T0 + Pichia kudriavzevii strain B-57; T7: T0 + *Pichia kudriavzevii* strain CBS 5147; T8: T0 + commercial yeast (*Saccharomyces cerevisiae*). Superscripts ^a,b,c,d,e^mean significantly different (p < 0.05).

Supplementation with lactic acid bacteria and yeast significantly increased (p < 0.05) the total gas production during the 48 h incubation period of rumen fermentation (Figure-1). Four of the five isolates of lactic acid bacteria produced higher total gas than the control (103 mL). The three yeast isolates showed higher total gas production than the control (103 mL). Supplementation with T6: *P. kudriavzevii* strain B-57 was shown to produce the highest total gas (183.33 mL) between the yeast candidate and the lactic acid bacteria candidates T4: *S. harbinensis* strain LH991 was shown to produce the highest total gas (149.17 mL). Treatments T4 and T6 showed results of total gas production that were substantiallydifferent from other treatments.

## Discussion

The microbes in the rumen environment are diverse. The selection of lactic acid bacteria and yeast strains as ruminant probiotic candidates were based on the digestibility of feed ingredients, rumen fermentation characteristics, and total gas production through *in vitro* fermentation. Probiotic supplementation should benefit the rumen environment through increased feed digestibility and decreased methane production [1]. The fermentability of the substrate may indicate that the microbes being supplemented, that is, lactic acid bacteria and yeast, can stimulate the microorganisms present in the rumen. This study showed that there was no significant change in the rumen pH when lactic acid bacteria and yeast were added (Table-2). All treatments showed a pH within the normal neutral range of 6.86–7.09. Because the pH conditions stayed within the normal range with the supplementation of lactic acid bacteria and yeast indicates the favorable rumen microbial activity. Lactic acid utilizing bacteria play a role in the utilization of lactate, thereby stabilizing the pH. This is corroborated with Seo *et al*. [8], who showed that in the rumen, there are lactic acid utilizing bacteria such as *Megasphaera elsdenii* and *Propionibacterium* spp., which utilize lactate to prevent its accumulation and maintaining the rumen pH. Propionibacteria can shift fermentation in the rumen by increasing propionate synthesis and propionate synthesis can reduce hydrogen in the production of enteric methane gas [24]. Yeast, as a feed additive for ruminants, can provide vitamins and organic acids to trigger the growth of lactic acid bacteria [25], maintain rumen pH stability, and increase anaerobiosis by consuming oxygen in the rumen [26]. Live yeast given to ruminants provides nutrition for bacteria that utilize lactic acid in the rumen to stabilize the pH and increase the growth of cellulolytic bacteria [27]. Weinberg *et al*. [28] demonstrated that a stable pH indicates that probiotics can survive in the rumen fluid and trigger the growth of rumen microbes, increasing feed digestibility and that rumen microorganisms can function properly [8, 29]. Rumen conditions, especially rumen pH, play a significant role in the capability to degrade protein feed by rumen microbes [30].

In this research, we observed an increase in the production of NH3 and total VFA in the supplementation with lactic acid bacteria and yeast in rumen fermentation *in vitro* (Table-2). Chen *et al*. [31] stated that probiotic supplementation was able to strengthen rumen fermentation. Volatile fatty acid is the end product of rumen fermentation, which is used by ruminants as the main contributor of energy [31]. The production and proportion of VFA can reflect the status of rumen microbial metabolism and determine the variety of fibrolytic or amylolytic microflora in the rumen, which plays a major role [31]. Based on these observations, the total VFA concentration in this research ranged from 81.67 mM to 121.67 mM, where the supplementation with lactic acid bacteria T4: *S. harbinensis* strain LH991 (121.67 mM) and T6: *P. kudriavzevii* strain B-5P (96.67 mM) showed the best results compared to the other treatments. These results are consistent with previous studies that showed that supplementation with *S. cerevisiae, Clostridium butyricum*, and their combinations could increase the total VFA concentrations [32, 33]. In line with the report of Jiao *et al*. [34] that the digestibility of dry matter and VFA increased with increasing supplementation of live yeast at pH 5.8. The increase in total VFA concentration by probiotic supplementation indicated that rumen microbe-stimulated fermentation activities [33] were ultimately able to increase livestock productivity [1].

The concentration of NH3 in this study ranged from 12.25 mM to 16.63 mM (Table-2). The best results shown by supplementation with candidate lactic acid bacteria were T4: *S. harbinensis* strain LH991 (16.83 mM) compared to supplementation with other lactic acid bacteria strains. As for the yeast candidate supplementation with T6: *P. kudriavzevii* strain B-5P (13.42 mM) showed the best results compared to the other yeast candidates. Microbial protein synthesis requires ammonia as the main source of N for microbes [35]. The production of NH3 in this study supported the synthesis of microbial proteins required by the ruminants during production. This is in line with the work by McDonald *et al*. [36] that showed that the concentration of NH3 levels of 6–21 mM can increase microbial protein production. About two-thirds of the amino acids absorbed by the ruminant’s body are produced from microbial proteins, a source of amino acids for ruminants [30]. Carbohydrates are the main source of energy needed for microbial protein synthesis [30]. The previous research by Zhang *et al*. [37] claimed that an increase in NH3 concentration occurs through supplementation with *C. butyricum* in batch culture using a high forage substrate and a pH 6.6 medium.

The selection of the best candidate strains of lactic acid bacteria and yeast strains from fermented fish (Budu) for ruminant probiotics was observed from the highest dry and organic matter’s digestibility and total gas production. *In vitro* measurement of total gas production can be used to evaluate the feed organic matter’s degradability (consisting of N and C sources) [38]. Meanwhile, gas production is positively related to the VFA results, but this relationship is not clearly understood [38]. Total gas production can be used as a consideration of digestibility in the rumen [39] and increased total gas production can be attributed to increased dry and OMD [1]. We observed an increase in total gas production (Figure-1), which was in agreement with the resulting digestibility (Table-3). Supplementation with lactic acid bacteria T4: *S. harbinensis* strain LH991 showed the highest dry and OMD (70.29% and 71.16%, respectively) as well as the highest total gas production (149.17 mL). The same applied to yeast strains when supplemented with T6: *P. kudriavzevii* strain B-5P that showed the highest dry and OMD (67.64% and 69.55%, respectively) as well as the highest total gas production (183.33 mL). Meanwhile, T1: *L. parabuchneri* strain 3347 supplementation resulted in the lowest digestibility of dry and organic matter (63.05% and 64.06%, respectively) and total gas production (91.67 mL), among other lactic acid treatments. The results of this investigation are consistent with the study by Guo *et al*. [40] where the increase in DMD was followed by an increase in the average gas production with the supplementation of lactic acid bacteria inoculums.

Increased feed digestibility indicates that probiotic supplementation with lactic acid bacteria and yeast from fermented fish (Budu) can trigger microbial activity in the rumen. Spores forming from the microbe probiotics can increase cellulolytic activity which supplies oligosaccharides to the beneficial microorganisms. Increased cellulolytic activity correlated with increased digestibility in ruminants [41]. Probiotics of live yeast can modulate the composition and activity of the microbial ecosystem increasing nutrient digestibility. In addition, yeast probiotics can stabilize rumen pH to activate fibrolytic bacteria in the rumen and increase fiber digestibility by probiotics [42]. In line with the work by Anee *et al*. [14], probiotic supplementation can increase the digestibility of food in ruminants. Astuti *et al*. [15] demonstrated that increased feed digestibility can confirm that lactic acid bacteria could be applied as a probiotic by stimulating the activity of rumen bacteria. Improved digestibility indicates that supplementing lactic acid bacteria can stimulate fibrolytic bacteria in the rumen [39]. This is similar to the addition of yeast culture as it affects the number of cellulolytic bacteria in the rumen thereby increasing the degradation of cellulose [42]. It has also been reported that *S. cerevisiae* supplementation stimulated the survival rate of cellulolytic bacteria, but not the fiber digestion rate of the rumen by its microorganisms [42]. This research agrees with the report by Al-Galbi and Majeed [43], where supplementation with *S. cerevisiae* increased the digestibility of dry matter and neutral detergent fiber. The digestibility of this feed can be increased with an increase in rumen microorganisms. In addition, *S. cerevisiae* can use oxygen to maintain metabolic activity. In another report by Jiao *et al*. [39], it was shown that lactic acid bacteria can increase feed degradation in the rumen, but it was dependent on the dose and strain used. Lactic acid bacteria can encourage rumen microbes to adapt to the presence of lactic acid and produce antimicrobials to reject pathogens [39]. The available lactic acid is used by lactate utilizing bacteria which consist of beneficial bacteria in nutrient degradation [44]. Lactic acid bacteria participate in the absorption and stabilization of fiber-degrading enzymes in the rumen to increase the degradation of feed components [45]. Meanwhile, other studies have reported that the supplementation with *Lactobacillus plantarum* as a probiotic did not significantly affect the digestibility of dry and organic matter [1].

## Conclusion

Probiotic supplementation with five lactic acid bacteria, two yeast isolates from fermented fish (Budu), and one commercial yeast isolate resulted in different digestibility levels and rumen fermentation. Lactic acid bacteria T4: *S. harbinensis* strain LH991 and yeast T6: *P. kudriavzevii* strain B-5P consistently showed the highest values of dry and OMD and total gas, VFA, and NH3 production. Lactic acid bacteria *S. harbinensis* strain LH991 and yeast *P. kudriavzevii* strain B-5P were identified as the best candidates for ruminant probiotics based on the digestibility of feed ingredients and rumen characteristics of *in vitro* fermentation. For future research, it is necessary to evaluate the total protozoa, microbial population, methane gas production, and composition of the resulting partial VFA.

## Authors’ Contributions

LRA: Conceptualized and designed the study, collected data, and drafted the manuscript. YM: Design formulated method for growing the inoculums probiotics. MZ: Design formulated in vitro material in the laboratory and supervised data. JJ: Processed and analyzed the data. DMF and JJ: Conducted sequencing and reading of microbial strains. LRA and DMF: Drafted, edited, and critically revised the manuscript. All authors have read, revised, and approved the final manuscript.

## References

[1] Astuti W.D, Wiryawan K.G, Wina E, Widyastuti Y, Suharti S, Ridwan R (2018). Effects of selected *Lactobacillus plantarum* as probiotic on *in vitro* ruminal fermentation and microbial population. Pak. J. Nutr.

[2] Hill C, Guarner F, Reid G, Gibson G.R, Merenstein D.J, Pot B, Morelli L, Canani R.B, Flint H.J, Salminen S, Calder P.C, Sanders M.E (2014). The international scientific association for probiotics and prebiotics consensus statement on the scope and appropriate use of the term probiotic. Nat. Rev. Gastroenterol. Hepatol.

[3] Uyeno Y, Shigemori S, Shimosato T (2015). Effect of probiotics/prebiotics on cattle health and productivity. Microbes Environ.

[4] Adjei-Fremah S, Ekwemalor K, Asiamah E.K, Ismail H, Ibrahim S, Worku M (2018). Effect of probiotic supplementation on growth and global gene expression in dairy cows. J. Appl. Anim. Res.

[5] Raabis S, Li W, Cersosimo L (2019). Effects and immune responses of probiotic treatment in ruminants. Vet. Immunol. Immunopathol.

[6] Naidu Y.Y, Rao K.A, Seshaiah C.V, Kumar D.S, Lekha M.S (2021). Effect of feeding multi-strain probiotic on milk quality and milk quantity in Murrah buffaloes. Int. J. Curr. Microbiol. Appl. Sci.

[7] Nalla K, Manda N.K, Dhillon H.S, Kanade S.R, Rokana N, Hess M, Puniya A.K (2022). Impact of probiotics on dairy production efficiency. Front. Microbiol.

[8] Seo J.K, Kim S.W, Kim M.H, Upadhaya S.D, Kam D.K, Ha J.K (2010). Direct-fed microbials for ruminant animals. Asian Aust. J. Anim. Sci.

[9] Nuraida L (2015). A review:Health promoting lactic acid bacteria in traditional Indonesian fermented foods. Food Sci. Hum. Wellness.

[10] Anggraini L, Marlida Y, Mirzah M, Wizna W, Jamsari J, Huda N (2019). Isolation and characterization of lactic acid bacteria producing GABA from indigenous West Sumatera fermented food. Int. J. Adv. Sci. Eng. Inf. Technol.

[11] Harnentis H, Marlida Y, Nur Y.S, Wizna W, Santi M.A, Septiani N, Adzitey F, Huda N (2020). Novel probiotic lactic acid bacteria isolated from indigenous fermented foods from West Sumatera, Indonesia. Vet. World.

[12] Efriwati E, Suwanto A, Rahayu G, Nuraida L (2013). Population dynamics of yeasts and lactic acid bacteria (LAB) during tempeh production. HAYATI J. Biosci.

[13] Huda N, Rosma A (2006). Budu and Tukai:Endemic fermented fish products from West Sumatra. INFOFISH Int.

[14] Anee I.J, Alam S, Begum R.A, Shahjahan R.M, Khandaker A.M (2021). The role of probiotics on animal health and nutrition. J. Basic Appl. Zool.

[15] Astuti W.D, Ridwan R, Fidriyanto R, Rohmatussolihat R, Sari N.F, Sarwono K.A, Fitri A, Widyastuti Y (2022). Changes in rumen fermentation and bacterial profiles after administering *Lactiplantibacillus plantarum* as a probiotic. Vet. World.

[16] Jiang X, Xu H.J, Cui Z.Q, Zhang Y.G (2020). Effects of supplementation with *Lactobacillus plantarum* 299v on the performance, blood metabolites, rumen fermentation and bacterial communities of preweaning calves. Livest. Sci.

[17] Harikrishna C, Mahender M, Reddy Y.R, Prakash M.G, Sudhakar K, Pavani M (2012). Evaluation of *in vitro* gas production and nutrient digestibility of complete diets supplemented with different levels of thermotolerant yeast in Nellore rams. Vet. World.

[18] Adeyemi J.A, Harmon D.L, Compart D.M.P, Ogunade I.M (2019). Effects of a blend of *Saccharomyces cerevisiae*-based direct-fed microbial and fermentation products in the diet of newly weaned beef steers:Growth performance, whole-blood immune gene expression, serum biochemistry, and plasma metabolome. J. Anim. Sci.

[19] Riyanti L, Suryahadi S, Evvyernie D (2016). *In vitro* fermentation characteristics and rumen microbial population of diet supplemented with *Saccharomyces cerevisiae* and rumen microbe probiotics. Media Peternakan.

[20] Sutardi ((1980)). Basic of Nutritional Science. Department of Animal Nutrition and Feed Science, Faculty of Animal Science.

[21] Tilley J.M.A, Terry R.A (1963). A two-stage technique for *in vitro* digestion of forage crops. J. Br. Grassl. Soc.

[22] Conway E.J, O'Malley E ((1942)). Microdiffusion methods Ammonia and urea using buffered absorbents (revised methods for ranges greater than 10 µg. N). Biochem. J.

[23] General Laboratory Procedures ((1996)). General Laboratory Procedures Department of Dairy Science.

[24] Chen J, Holo H, Schwarm A, Harstad O.M (2020). Ruminal survival of *Propionibacterium thoenii* T159 in dairy cows at high feed intake. Acta Agric. Scand. A Anim. Sci.

[25] Arowolo M.A, He J (2018). Use of probiotics and botanical extracts to improve ruminant production in the tropics:A review. Anim. Nutr.

[26] Khan R.U, Naz S, Dharma K, Karthik K, Tiwari R, Abdelrahman M.M, Alhidary I.A, Zahoor A (2016). Direct-fed microbial:Beneficial applications, modes of action and prospects as a safe tool for enhancing ruminant production and safeguarding health. Int. J. Pharmacol.

[27] Peng Q.H, Cheng L, Kang K, Tian G, Al-Mamun M, Xue B, Wang L.Z, Zou H.W, Gichena M.G, Wang Z.S (2020). Effects of yeast and yeast cell wall polysaccharides supplementation on beef cattle growth performance, rumen microbial populations and lipopolysaccharides production. J. Integr. Agric.

[28] Weinberg Z.G, Shatz O, Chen Y, Yosef E, Nikbahat M, Ben-Ghedalia D, Miron J (2007). Effect of lactic acid bacteria inoculants on *in vitro* digestibility of wheat and corn silages. J. Dairy Sci.

[29] Ridwan R, Bungsu W.A, Astuti W.D, Rohmatussolihat R, Sari N.F, Fidriyanto R, Jayanegara A, Wijayanti I, Widyastuti Y (2018). The use of lactic acid bacteria as a ruminant probiotic candidates based on *in vitro* rumen fermentation characteristics. Buletin Peternakan.

[30] Pathak A.K (2008). Various factors affecting microbial protein synthesis in the rumen. Vet. World.

[31] Chen Y.Y, Wang Y.L, Wang W.K, Zhang Z.W, Si X.M, Cao Z.J, Li S.L, Yang H.J (2020). Beneficial effect of *Rhodopseudomonas palustris* on *in vitro* rumen digestion and fermentation. Benef. Microbes.

[32] Cai L, Yu J, Hartanto R, Qi D (2021). Dietary supplementation with *Saccharomyces cerevisiae*,*Clostridium butyricum* and their combination ameliorate rumen fermentation and growth performance of heat-stressed goats. Animals (Basel).

[33] Mao H.L, Mao H.L, Wang J.K, Liu J.X, Yoon I (2013). Effects of *Saccharomyces cerevisiae* fermentation product on *in vitro* fermentation and microbial communities of low-quality forages and mixed diets. J. Anim. Sci.

[34] Jiao P, Wei C, Sun Y, Xie X, Zhang Y, Wang S, Hu G, Alzahal O, Yang W (2019). Screening of live yeast and yeast derivatives for their impact of strain and dose on *in vitro* ruminal fermentation and microbial profiles with varying media pH levels in high-forage beef cattle diet. J. Sci. Food. Agric.

[35] Bach A, Calsamiglia S, Stern M.D (2005). Nitrogen metabolism in the rumen. J. Dairy Sci.

[36] McDonald P, Edwards R.A, Greenhalgh J.F.D, Morgan C.A (2002). Animal Nutrition.

[37] Zhang M, Liang G, Zhang X, Lu X, Li S, Wang X, Yang W, Yuan Y, Jiao P (2022). The gas production, ruminal fermentation parameters, and microbiota in response to *Clostridium butyricum* supplementation on *in vitro* varying with media pH levels. Front. Microbiol.

[38] Contreras-Govea F.E, Muck R.E, Mertens D.R, Weimer P.J (2011). Microbial inoculant effects on silage and *in vitro* ruminal fermentation, and microbial biomass estimation for alfalfa, BMR corn, and corn silages. Anim. Feed Sci. Technol.

[39] Jiao P.X, Liu F.Z, Beauchemin K.A, Yang W.Z (2017). Impact of strain and dose of lactic acid bacteria on *in vitro* ruminal fermentation with varying media pH levels and feed substrates. Anim. Feed Sci. Technol.

[40] Guo G, Shen C, Liu Q, Shang S.L, Shao T, Wang C, Wang Y.X, Xu Q.F, Huo W.J (2020). The effect of lactic acid bacteria inoculums on *in vitro* rumen fermentation, methane production, ruminal cellulolytic bacteria populations and cellulase activities of corn stover silage. J. Integr. Agric.

[41] Qiao G.H, Shan. A.S Ma, N, Ma Q.Q, Sun Z.W (2010). Effect of supplemental *Bacillus* cultures on rumen fermentation and milk yield in Chinese Holstein cows. J. Anim. Physiol. Anim. Nutr (Berl).

[42] Dehghan-Banadaky M, Ebrahimi M, Motameny R, Heidari S.R (2013). Effects of live yeast supplementation on mid-lactation dairy cows performances, milk composition, rumen digestion and plasma metabolites during hot season. J. Appl. Anim Res.

[43] Al-Galbi H.A.J, Majeed M.S (2022). Effect of concentrate:Roughage ratio and the addition of Kefir on the production characteristics of ruminant *in vitro*. Arch. Razi Inst.

[44] Nocek J.E, Kautz W.P, Leedle J.A.Z, Allman J.G (2022). Ruminal supplementation of direct-fed microbials on diurnal pH variation and in situ digestion in dairy cattle. J. Dairy Sci.

[45] Schofield B.J, Lachner N, Le O.T, McNeill D.M, Dart P, Ouwerkerk D, Hugenholtz P, Klieve A.V (2018). Beneficial changes in rumen bacterial community profile in sheep and dairy calves as a result of feeding the probiotic *Bacillus amyloliquefaciens* H57. J. Appl. Microbiol.

